# Cytokeratin 5 and cytokeratin 6 expressions are unconnected in normal and cancerous tissues and have separate diagnostic implications

**DOI:** 10.1007/s00428-021-03204-4

**Published:** 2021-09-24

**Authors:** Cosima Völkel, Noémi De Wispelaere, Sören Weidemann, Natalia Gorbokon, Maximilian Lennartz, Andreas M. Luebke, Claudia Hube-Magg, Martina Kluth, Christoph Fraune, Katharina Möller, Christian Bernreuther, Patrick Lebok, Till S. Clauditz, Frank Jacobsen, Guido Sauter, Ria Uhlig, Waldemar Wilczak, Stefan Steurer, Sarah Minner, Rainer H. Krech, David Dum, Till Krech, Andreas H. Marx, Ronald Simon, Eike Burandt, Anne Menz

**Affiliations:** 1 Institute of Pathology, University Medical Center Hamburg-Eppendorf, Martinistr. 52, 20246 Hamburg, Germany; 2 General, Visceral and Thoracic Surgery Department and Clinic, University Medical Center Hamburg-Eppendorf, Hamburg, Germany; 3 Institute of Pathology, Clinical Center Osnabrueck, Osnabrueck, Germany; 4 Department of Pathology, Academic Hospital Fuerth, Fuerth, Germany

**Keywords:** Cytokeratin 5, Cytokeratin 6, Tissue microarray, Immunohistochemistry, Diagnostic

## Abstract

Cytokeratins (CKs) 5 and 6 are functionally unrelated but often analyzed together using bispecific antibodies in diagnostic immunohistochemistry. To better understand the diagnostic utility of CK5 or CK6 alone, tissue microarrays with > 15,000 samples from 120 different tumor types as well as 608 samples of 76 different normal tissues were analyzed by immunohistochemistry. In normal tissues, both CKs occurred in the squamous epithelium; CK5 dominated in basal and CK6 in suprabasal layers. CK5 (not CK6) stained basal cells in various other organs. Within tumors, both CK5 and CK6 were seen in > 95% of squamous cell carcinomas, but other tumor entities showed different results: CK5 predominated in urothelial carcinoma and mesothelioma, but CK6 in adenocarcinomas. Joint analysis of both CK5 and CK6 obscured the discrimination of epithelioid mesothelioma (100% positive for CK5 alone and for CK5/6) from adenocarcinoma of the lung (12.8% positive for CK5 alone; 23.7% positive for CK5/6). CK5 and CK6 expressions were both linked to high grade, estrogen receptor, and progesterone receptor negativity in breast cancer (*p* < 0.0001 each), grade/stage progression in urothelial cancer (*p* < 0.0001), and RAS mutations in colorectal cancer (*p* < 0.01). Useful diagnostic properties which are commonly attributed to CK5/6 antibodies such as basal cell staining in the prostate, distinction of adenocarcinoma of the lung from squamous cell carcinoma and epithelioid mesothelioma, and identification of basal-type features in urothelial cancer are solely driven by CK5. At least for the purpose of distinguishing thoracic tumors, monospecific CK5 antibodies may be better suited than bispecific CK5/6 antibodies.

**Supplementary Information:**

The online version contains supplementary material available at 10.1007/s00428-021-03204-4.

## Introduction

Cytokeratins 5 and 6 are basic type II cytokeratins which are not functionally related [1]. Cytokeratin 5 (CK5) forms heterodimers with cytokeratin 14, and cytokeratin 6 (CK6) forms heterodimers with cytokeratin 16 [2, 3]. However, cytokeratins 5 and 6 are often jointly examined by immunohistochemistry because common antibodies recognize both cytokeratins 5 and 6 and the use of these bispecific antibodies has clinical utility [4–6]. Cytokeratin 5/6 (CK5/CK6) antibodies are for example applied to identify basal cells or myoepithelial cells for ruling out invasive breast and prostate cancer, to detect squamous cell origin in poorly differentiated carcinomas [6], and to distinguish epithelioid mesothelioma (mostly CK5/6 positive) from lung adenocarcinoma (mostly CK5/6 negative) [5]. CK5/6 immunostaining has also been proposed to have prognostic utility in triple-negative breast cancer [7–9], urothelial carcinoma [10–12], and other tumors [13–15].

Most previous immunohistochemical studies on the diagnostic and prognostic role of CK5 and CK6 have employed antibodies directed against both proteins. Studies analyzing either CK5 or CK6 alone are limited to less than 100 but the results were still partly conflicting. For example, CK5 positivity has been described in 13.6 to 91% of bladder carcinomas [11, 16], 2.5 to 100% of breast carcinomas [17, 18], 59.5 to 100% of head and neck carcinoma [14, 19], 0 to 100% of lung carcinomas [20–25] and 74.8 to 93.8% of mesothelioma carcinomas [26–28]. Even less is known about CK6 positivity alone, which has been reported to occur in 18% of endometrial stromal sarcomas [29], 28% of gastric cancers [30], 38% of basal cell carcinomas of the skin [31], and 100% of squamous cell cancers of the head and neck [32, 33].

To better understand the clinical utility of immunohistochemical analysis of CK5 and CK6 alone, both proteins were analyzed in more than 14,000 tumor tissue samples from 120 different tumor types and subtypes as well as 76 non-neoplastic tissue categories by immunohistochemistry (IHC) in a tissue microarray (TMA) format in this study.

## Materials and methods

### Tissue microarrays (TMAs)

Our normal tissue TMA was composed of 8 samples from 8 different donors for each of 76 different normal tissue types (608 samples on one slide). The cancer TMAs contained a total of 15,966 primary tumors from 120 tumor types and subtypes. Histopathological data on pathological tumor stage (pT), histological grade, and pathological lymph node status (pN) were available from up to 2,075 breast, 1,663 bladder, 327 gastric, 598 pancreatic, 2,351 colorectal, 524 ovarian, and 259 endometrial cancers. Clinical follow-up data were available from 1,183 breast cancer and 254 urinary bladder cancer patients with a median follow-up time of 49/14 months (range 1–88/1–77). Molecular data on HER2, estrogen receptor (ER), and progesterone receptor (PR) status, microsatellite instability (MSI), and RAS mutations were available from previous studies [34, 35]. The composition of both normal and cancer TMAs is described in the results section. All samples were from the archives of the Institutes of Pathology, University Hospital of Hamburg, Germany, the Institute of Pathology, Clinical Center Osnabrueck, Germany, and Department of Pathology, Academic Hospital Fuerth, Germany. Tissues were fixed in 4% buffered formalin and then embedded in paraffin. The TMA manufacturing process was described earlier in detail [36, 37]. In brief, one tissue spot (diameter: 0.6 mm) was transmitted from a representative cancer-containing donor block in an empty recipient paraffin block. The use of archived remnants of diagnostic tissues for manufacturing of TMAs and their analysis for research purposes as well as patient data analysis were according to local laws (HmbKHG, §12) and approved by the local ethics committee (Ethics Commission Hamburg, WF-049/09). All work has been carried out in compliance with the Helsinki declaration.

### Immunohistochemistry

Freshly cut TMA sections were immunostained on 1 day and in one experiment. Slides were deparaffinized with xylol, rehydrated through a graded alcohol series, and exposed to heat-induced antigen retrieval for 5 min in an autoclave at 121 °C in pH 9 DakoTarget Retrieval Solution™ (Agilent, CA, USA; #S2367,). Endogenous peroxidase activity was blocked with Dako Peroxidase Blocking Solution™ (Agilent, CA, USA; #52,023) for 10 min. Primary antibodies specific for CK5 protein (mouse monoclonal, clone MSVA-605 M) and CK6 protein (rabbit recombinant, clone MSVA-606R), both from MS Validated Antibodies, Hamburg, Germany were applied at a dilution of 1:150 each at 37 °C for 60 min. Bound antibody was then visualized using the EnVision Kit™ (Agilent, CA, USA; #K5007) according to the manufacturer’s directions. For tumor tissues, the percentage of positive neoplastic cells was estimated, and the staining intensity was semiquantitatively recorded (0, 1 + , 2 + , 3 +). For statistical analyses, the staining results were categorized into four groups. Tumors without any staining were considered negative. Tumors with 1 + staining intensity in ≤ 70% of cells or 2 + intensity in ≤ 30% of cells were considered weakly positive. Tumors with 1 + staining intensity in > 70% of cells or 2 + intensity in 31–70% or 3 + intensity in ≤ 30% were considered moderately positive. Tumors with 2 + intensity in > 70% or 3 + intensity in > 30% of cells were considered strongly positive.

### Statistics

Statistical calculations were performed with JMP 14 software (SAS Institute Inc., NC, USA). Contingency tables and the chi^2^-test were performed to search for associations between CK5 or CK6 and tumor phenotype. Survival curves were calculated according to Kaplan–Meier. The Log-Rank test was applied to detect significant differences between groups. A *p*-value of ≥ 0.05 was considered as statistically significant.

## Results

### Technical issues

A total of 12,525 (78.5%) of 15,966 tumor samples were interpretable for CK5 and 12,898 (80.8%) of 15,966 tumor samples were interpretable for CK6 in this TMA analysis. The remaining samples were not analyzable due to the lack of unequivocal tumor cells or loss of the tissue spot during the technical procedures. In the normal tissue TMA, a sufficient number of samples was always interpretable per tissue type to determine CK5 and CK6 expressions.

### CK5 in normal tissues

**Fig. 1:**
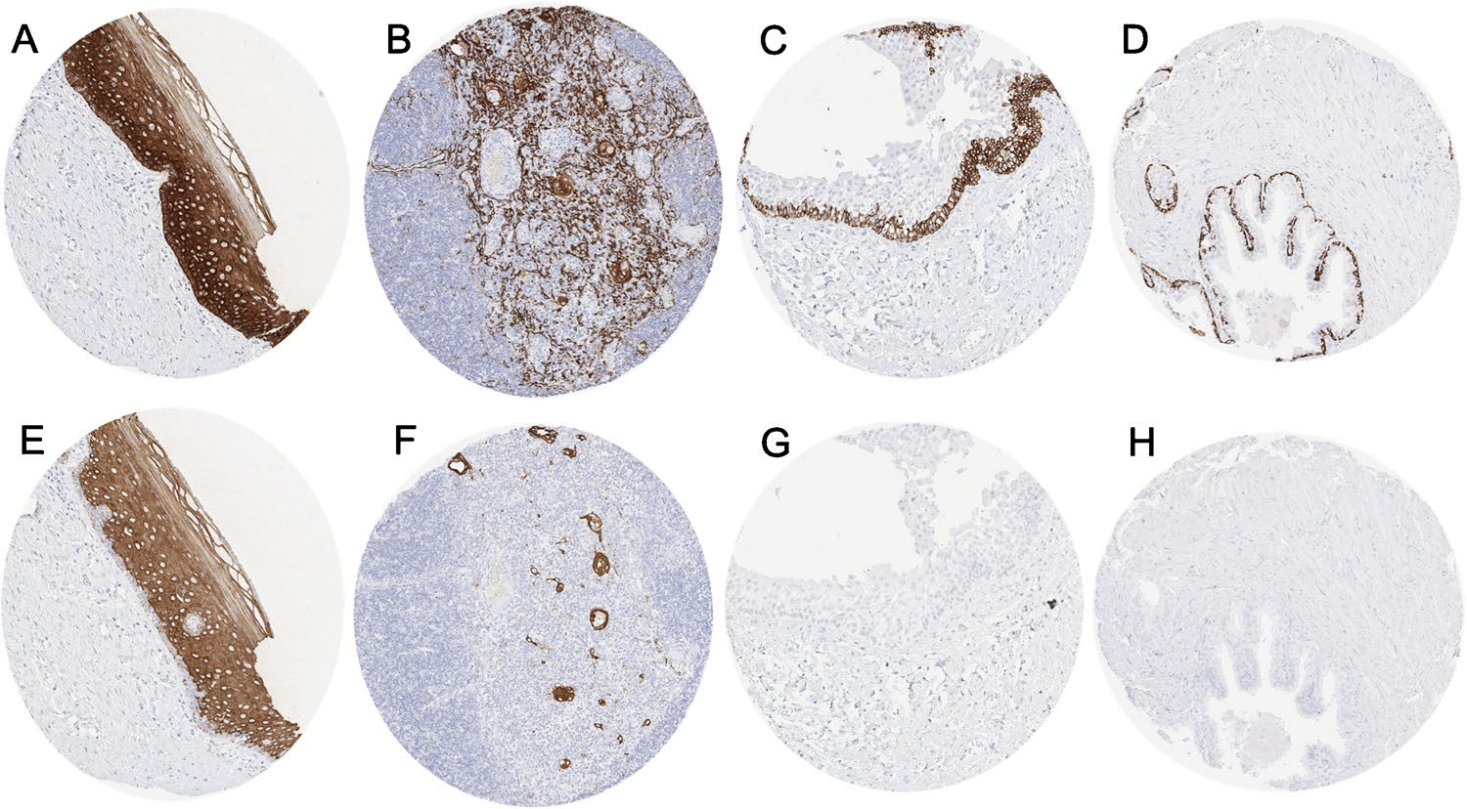
CK5 and CK6 immunostaining of normal tissues. The panels show for CK5 a strong staining of all cell layers of squamous epithelium of the uterine cervix (**A**), virtually all thymic epithelial cells (**B**), the basal cell layer of the urothelium (**C**), and prostate gland basal cells (**D**). Although CK6 immunostaining did often involve similar tissues as seen for CK5, CK6 staining was largely lacking in the basal cell layer of the squamous epithelium of the uterine cervix (**E**), thymic epithelial cells except corpuscles of Hassal’s (**F**), the urothelium (**G**), and basal cells of the prostate (**H**). The images **A**–**D** and **E**–**H** were taken from consecutive tissue sections

Strong CK5 immunostaining was seen in all keratinizing and non-keratinizing squamous epithelia (Fig. 1A) with a predominance of the staining in the basal cells of the epidermis, hair follicles, sebaceous glands, all epithelial cells of tonsil crypts, and of the thymus (Fig. 1B). In the urothelium, only the basal cell layers stained CK5 positive (Fig. 1C). CK5 immunostaining was also seen in myoepithelial cells and basal cells of excretion ducts of salivary and bronchial glands, basal cells of the prostate (Fig. 1D), seminal vesicle, respiratory epithelium, endocervix (not all glands), columnar cells (not all), and basal cells of the epididymis, myoepithelial cells of the breast. Amnion and chorion cells of the placenta also showed strong CK5 staining. CK5 immunostaining was absent in the lung, liver, pancreas, testis, kidney, gastrointestinal epithelial cells, Brunner glands, fallopian tube, adrenal gland, parathyroid gland, brain, adeno- and neurohypophysis, spleen, lymph node, all hematopoetic cell types, and all mesenchymal tissues.

### CK6 in normal tissues

A preferential immunostaining in squamous epithelial cells was also seen for CK6 but the staining was most intense in suprabasal cell layers and basal cells were negative or only weakly stained (Fig. 1E). A strong CK6 positivity was also seen in hair follicles, sebaceous glands, a fraction of the squamous cells in tonsil crypts, corpuscles of Hassall’s (but not in other epithelial cells) of the thymus (Fig. 1F), intercalated ducts of salivary and bronchial glands, basal cells and sometimes also ciliated cells (but not ciliae) of respiratory epithelium, few scattered endometrial cells (only in few samples), and in amnion/chorion cells (but not trophoblastic cells) of the placenta. CK6 immunostaining was absent in the urothelium (Fig. 1G), lung, liver, pancreas, prostate (Fig. 1H), seminal vesicle, epididymis, testis, kidney, gastrointestinal epithelial cells, Brunner glands, fallopian tube, adrenal gland, thyroid, parathyroid gland, brain, adeno- and neurohypophysis, spleen, lymph node, all hematopoietic cell types, and all mesenchymal tissues.

### CK5 and CK6 in tumors

**Fig. 2:**
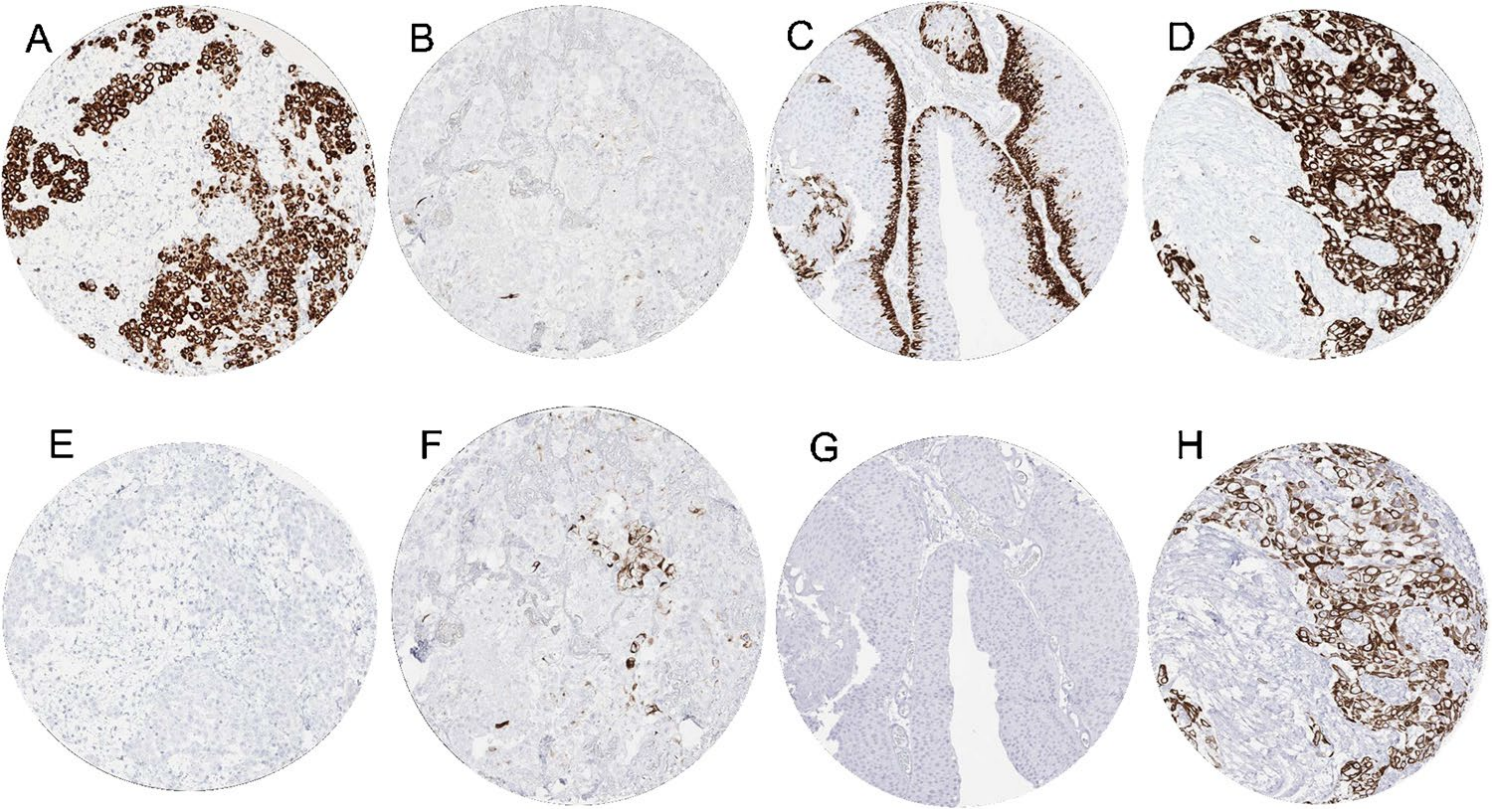
CK5 and CK6 immunostaining in cancer. For CK5, the panels show a strong staining of all epithelial cells of a malignant mesothelioma (**A**), a lack of staining in an adenocarcinoma of the lung (**B**), a “basal cell type” staining pattern in a non-invasive papillary (grade 2) urothelial carcinoma (**C**), and a diffuse positivity of all cells of an invasive urothelial carcinoma (**D**). The panels **E**–**H** show CK6 staining of consecutive tissue sections of the samples **A**–**D**. They show a lack of CK6 staining in a malignant mesothelioma (**E**), a focal positivity in an adenocarcinoma of the lung (**F**), absence of staining in a non-invasive papillary urothelial carcinoma (**G**), and a diffuse positivity of all cells of an invasive urothelial carcinoma (**H**)

**Fig. 3:**
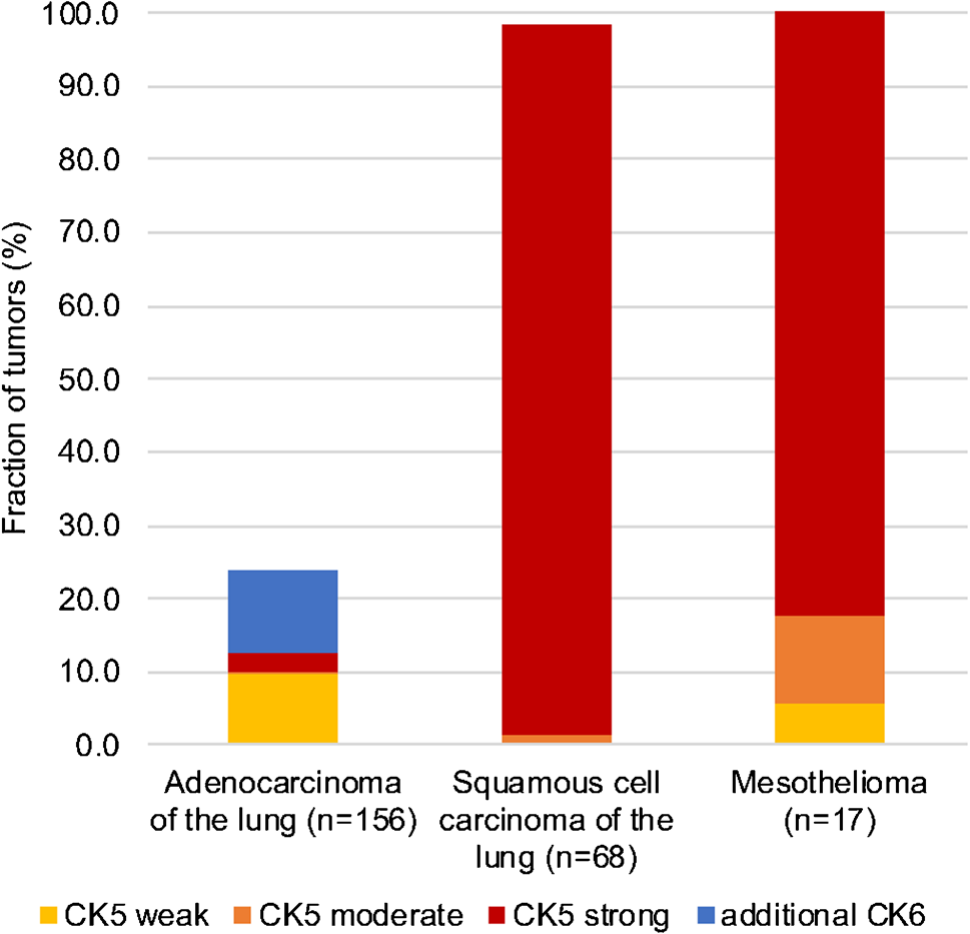
Obstructive role of CK6 for differential diagnosis of lung adenocarcinomas and lung squamous cell cancers or mesotheliomas

Our tumor analysis revealed CK5 positivity in 21.1% (4.4% weak, 2.7% moderate, 14.0% strong) and CK6 positivity in 22.8% (8.4% weak, 4.1% moderate, 10.4% strong) of tumors. Of 2,920 CK5 and/or CK6 positive tumors, 1,921 (66%) showed staining for both CK5 and CK6, 461 (16%) stained positive only for CK5, and 538 (18%) showed positivity for CK6 alone. A detailed description of the results for CK5 and CK6 is given in Table 1. Representative images are shown in Fig. 2. Both antibodies showed positive staining in > 95% of squamous cell carcinomas from various origins. For most other tumor entities, CK5 and CK6 showed different positivity rates. CK5 was the predominant staining in mesothelioma, basal cell carcinoma of the skin, urothelial carcinoma, thymoma, and salivary gland tumors, while CK6 predominated in various adenocarcinomas. It was noticeable for all tumor entities that either CK5 or CK6 was the predominant staining and that adding the second cytokeratin did not relevantly increase the fraction of positive cases (Supplementary Fig. 1). A separate analysis of thoracic tumors (mesothelioma vs. squamous cell carcinoma of the lung vs. adenocarcinoma of the lung) revealed that the combined use of both CK5 and CK6 immunostaining hindered the discrimination of these tumors because the positivity rate of lung adenocarcinomas increased from 12.8% (CK5 alone) to 23.7% (CK5 and/or CK6 positive) while both squamous cell carcinomas and epithelioid mesotheliomas were positive in 100% irrespective of whether CK5 alone or CK5 and CK6 were jointly applied (Fig. 3).

**Table 1:** CK5, CK6, and CK5/CK5 immunostaining in human tumors (*n analyzable for CK5 and CK6; *w*, weak; *m*, moderate; *s*, strong; *neg*, negative; *pos* positive)

**Tumor entity**			**CK5**			**CK6**			**CK5/CK6**		
	**on TMA (*n*)**	n*	**w (%)**	**m (%)**	**s (%)**	**w (%)**	**m (%)**	**s (%)**	**CK5 neg / CK6 pos**	**CK5 pos / CK6 neg**	**CK5 pos / CK6 pos**
**Tumors of the skin**											
Pilomatrixoma	35	22	8.7	26.1	8.7	9.7	12.9	0.0	0.0	18.2	22.7
Basal cell carcinoma	88	37	0.0	0.0	100.0	41.8	44.8	7.5	0.0	10.8	89.2
Benign nevus	29	25	0.0	0.0	0.0	0.0	0.0	0.0	0.0	0.0	0.0
Squamous cell carcinoma of the skin	90	86	0.0	1.1	97.7	1.2	0.0	96.5	0.0	1.2	97.7
Malignant melanoma	48	43	0.0	0.0	0.0	0.0	0.0	0.0	0.0	0.0	0.0
Merkel cell carcinoma	46	31	6.5	0.0	0.0	0.0	0.0	0.0	0.0	6.5	0.0
**Tumors of the head and neck**											
Squamous cell carcinoma of the larynx	110	95	1.0	2.0	96.0	3.1	2.1	92.8	1.1	2.1	96.8
Squamous cell carcinoma of the pharynx	60	51	1.8	0.0	96.4	3.6	12.5	83.9	2.0	0.0	98.0
Oral squamous cell carcinoma (floor of the mouth)	130	120	1.6	0.8	96.0	4.8	5.6	88.0	0.8	0.8	97.5
Pleomorphic adenoma of the parotid gland	50	20	6.1	6.1	72.7	25.8	25.8	0.0	5.0	45.0	35.0
Warthin tumor of the parotid gland	49	39	0.0	52.1	47.9	56.3	33.3	6.3	0.0	5.1	94.9
Basal cell adenoma of the salivary gland	15	10	6.7	0.0	93.3	26.7	33.3	20.0	0.0	30.0	70.0
**Tumors of the lung, pleura, and thymus**											
Adenocarcinoma of the lung	246	156	9.1	3.6	2.4	16.9	5.6	6.8	10.9	0.0	12.8
Squamous cell carcinoma of the lung	130	68	0.0	1.4	97.3	2.8	4.2	87.5	0.0	1.5	97.1
Small cell carcinoma of the lung	20	14	7.1	7.1	0.0	0.0	14.3	0.0	0.0	0.0	14.3
Mesothelioma, epithelioid	39	17	5.3	10.5	84.2	20.0	10.0	20.0	0.0	52.9	47.1
Mesothelioma, other types	76	35	5.3	7.9	52.6	39.1	0.0	0.0	2.9	22.9	40.0
Thymoma	29	21	0.0	3.7	92.6	15.4	19.2	3.8	0.0	76.2	23.8
**Tumors of the female genital tract**											
Squamous cell carcinoma of the vagina	78	66	1.4	0.0	98.6	5.8	4.3	88.4	0.0	0.0	100.0
Squamous cell carcinoma of the vulva	130	116	0.0	0.8	99.2	0.8	2.5	96.6	0.0	0.0	100.0
Squamous cell carcinoma of the cervix	130	111	0.0	2.5	95.0	1.7	8.3	86.8	0.0	0.0	97.3
Endometrioid endometrial carcinoma	236	183	23.8	11.6	4.2	15.6	14.2	2.2	3.8	14.2	24.6
Endometrial serous carcinoma	82	55	16.9	6.8	0.0	14.7	8.8	1.5	7.3	7.3	18.2
Carcinosarcoma of the uterus	48	37	21.1	10.5	13.2	13.0	15.2	2.2	0.0	16.2	27.0
Endometrial carcinoma, high grade, G3	13	11	8.3	8.3	8.3	8.3	8.3	8.3	0.0	9.1	18.2
Endometrial clear cell carcinoma	8	4	25.0	25.0	0.0	50.0	12.5	0.0	0.0	0.0	50.0
Endometrioid carcinoma of the ovary	110	72	25.3	8.9	8.9	20.3	10.1	2.5	5.6	18.1	25.0
Serous carcinoma of the ovary	559	355	23.6	6.2	8.3	32.6	5.8	1.1	12.1	13.2	25.1
Mucinous carcinoma of the ovary	96	69	2.8	1.4	2.8	7.0	1.4	1.4	4.3	1.4	4.3
Clear cell carcinoma of the ovary	50	37	5.3	2.6	0.0	17.9	0.0	0.0	16.2	5.4	2.7
Carcinosarcoma of the ovary	47	29	3.2	9.7	9.7	7.0	4.7	7.0	3.4	10.3	10.3
Brenner tumor	9	8	12.5	37.5	25.0	0.0	0.0	12.5	0.0	62.5	12.5
**Tumors of the breast**											
Invasive breast carcinoma of no special type	1391	930	3.9	1.7	4.4	7.8	6.1	3.4	8.1	1.7	8.0
Lobular carcinoma of the breast	294	171	1.6	0.0	1.6	3.7	1.6	1.1	2.3	0.6	2.3
Medullary carcinoma of the breast	26	18	20.0	0.0	20.0	0.0	9.1	4.5	0.0	33.3	5.6
Tubular carcinoma of the breast	27	13	13.3	0.0	0.0	0.0	0.0	0.0	0.0	15.4	0.0
Mucinous carcinoma of the breast	58	37	2.6	0.0	0.0	2.6	0.0	0.0	0.0	0.0	2.7
Phyllodes tumor of the breast	50	41	23.3	11.6	9.3	26.1	6.5	4.3	9.8	17.1	26.8
**Tumors of the digestive system**											
Adenomatous polyp, low-grade dysplasia	50	38	0.0	0.0	0.0	10.5	2.6	0.0	13.2	0.0	0.0
Adenomatous polyp, high-grade dysplasia	50	47	0.0	0.0	0.0	6.4	0.0	0.0	6.4	0.0	0.0
Adenocarcinoma of the colon	1932	1520	2.9	1.1	0.4	13.4	3.6	1.9	12.2	0.3	3.8
Gastric adenocarcinoma, diffuse type	226	120	0.0	0.0	0.7	4.5	1.5	2.3	8.3	0.0	0.8
Gastric adenocarcinoma, intestinal type	224	142	7.3	4.6	4.6	12.6	7.9	7.9	12.0	1.4	14.1
Gastric adenocarcinoma, mixed type	62	39	2.3	2.3	0.0	11.4	9.1	4.5	20.5	0.0	2.6
Adenocarcinoma of the esophagus	133	75	5.0	5.0	1.3	11.5	3.8	5.1	8.0	0.0	10.7
Squamous cell carcinoma of the esophagus	124	58	1.5	1.5	94.1	4.5	10.6	81.8	0.0	0.0	98.3
Squamous cell carcinoma of the anal canal	91	68	0.0	1.4	97.3	5.5	4.1	90.4	0.0	0.0	100.0
Cholangiocarcinoma	114	96	7.8	3.9	6.8	7.7	4.8	9.6	4.2	1.0	14.6
Hepatocellular carcinoma	50	47	0.0	0.0	0.0	0.0	0.0	0.0	0.0	0.0	0.0
Ductal adenocarcinoma of the pancreas	662	447	12.5	5.7	14.5	27.4	13.7	16.3	20.4	0.2	31.3
Pancreatic/Ampullary adenocarcinoma	119	77	17.9	5.1	5.1	24.4	8.5	4.9	13.0	5.2	22.1
Acinar cell carcinoma of the pancreas	16	15	0.0	0.0	0.0	0.0	0.0	0.0	0.0	0.0	0.0
Gastrointestinal stromal tumor (GIST)	50	47	0.0	0.0	0.0	0.0	0.0	0.0	0.0	0.0	0.0
**Tumors of the urinary system**											
Non-invasive papillary urothelial ca., pTa G2 low grade	177	115	31.1	23.8	32.8	6.7	4.5	2.2	0.0	75.7	12.2
Non-invasive papillary urothelial ca., pTa G2 high grade	141	85	27.8	25.6	18.9	14.4	7.7	2.9	0.0	48.2	22.4
Non-invasive papillary urothelial ca., pTa G3	187	85	17.4	17.4	14.1	14.7	3.9	2.0	1.2	29.4	18.8
Urothelial carcinoma, pT2-4 G3	1207	635	7.9	7.3	43.8	13.6	5.5	27.9	1.7	12.9	45.5
Small cell neuroendocrine carcinoma of the bladder	18	13	0.0	0.0	0.0	0.0	6.3	0.0	0.0	0.0	0.0
Clear cell renal cell carcinoma	1226	794	0.0	0.1	0.0	0.0	0.0	0.0	0.0	0.1	0.0
Papillary renal cell carcinoma	320	220	0.0	0.0	0.0	0.0	0.0	0.0	0.0	0.0	0.0
Clear cell (tubulo) papillary renal cell carcinoma	28	18	0.0	0.0	0.0	0.0	0.0	0.0	0.0	0.0	0.0
Chromophobe renal cell carcinoma	151	102	0.0	0.0	0.0	0.0	0.0	0.0	0.0	0.0	0.0
Oncocytoma	199	145	0.0	0.0	0.0	0.0	0.0	0.0	0.0	0.0	0.0
**Tumors of the male genital organs**											
Adenocarcinoma of the prostate, Gleason 3 + 3	83	83	0.0	0.0	0.0	0.0	0.0	0.0	0.0	0.0	0.0
Adenocarcinoma of the prostate, Gleason 4 + 4	80	80	0.0	0.0	0.0	1.3	0.0	0.0	1.3	0.0	0.0
Adenocarcinoma of the prostate, Gleason 5 + 5	85	84	0.0	0.0	0.0	0.0	0.0	0.0	0.0	0.0	0.0
Adenocarcinoma of the prostate (recurrence)	261	244	0.0	0.0	0.4	0.0	1.2	0.0	0.8	0.0	0.0
Small cell neuroendocrine carcinoma of the prostate	17	12	0.0	0.0	0.0	0.0	0.0	0.0	0.0	0.0	0.0
Seminoma	621	594	0.0	0.0	0.0	0.0	0.0	0.0	0.0	0.0	0.0
Embryonal carcinoma of the testis	50	21	0.0	0.0	0.0	0.0	0.0	0.0	0.0	0.0	0.0
Yolk sac tumor	50	32	0.0	0.0	0.0	3.0	0.0	0.0	3.1	0.0	0.0
Teratoma	50	20	3.7	7.4	25.9	7.4	11.1	11.1	0.0	15.0	15.0
Squamous cell carcinoma of the penis	80	77	0.0	0.0	100.0	3.8	1.3	94.9	0.0	0.0	100.0
**Tumors of endocrine organs**											
Adenoma of the thyroid gland	114	108	0.0	0.0	0.0	0.0	0.0	0.0	0.0	0.0	0.0
Papillary thyroid carcinoma	392	365	0.0	0.0	0.0	1.1	0.5	0.0	1.1	0.0	0.0
Follicular thyroid carcinoma	158	144	0.0	0.0	0.0	0.0	0.0	0.7	0.0	0.0	0.0
Medullary thyroid carcinoma	107	95	0.0	0.0	0.0	0.0	0.0	0.0	0.0	0.0	0.0
Anaplastic thyroid carcinoma	45	40	2.4	0.0	17.1	0.0	0.0	17.1	0.0	2.5	17.5
Adrenal cortical adenoma	50	27	0.0	0.0	0.0	0.0	0.0	0.0	0.0	0.0	0.0
Adrenal cortical carcinoma	26	23	0.0	0.0	0.0	0.0	0.0	0.0	0.0	0.0	0.0
Phaeochromocytoma	50	43	0.0	0.0	0.0	0.0	0.0	0.0	0.0	0.0	0.0
Appendix, neuroendocrine tumor (NET)	22	11	8.3	0.0	0.0	0.0	0.0	0.0	0.0	0.0	0.0
Colorectal, neuroendocrine tumor (NET)	11	11	0.0	0.0	0.0	0.0	0.0	0.0	0.0	0.0	0.0
Ileum, neuroendocrine tumor (NET)	49	41	0.0	0.0	0.0	0.0	0.0	0.0	0.0	0.0	0.0
Lung, neuroendocrine tumor (NET)	19	18	5.6	0.0	0.0	5.6	0.0	0.0	5.6	5.6	0.0
Pancreas, neuroendocrine tumor (NET)	98	79	0.0	0.0	1.2	1.3	3.8	0.0	3.8	1.3	0.0
Colorectal, neuroendocrine carcinoma (NEC)	12	7	0.0	0.0	10.0	0.0	0.0	0.0	0.0	14.3	0.0
Gallbladder, neuroendocrine carcinoma (NEC)	4	4	0.0	0.0	0.0	0.0	0.0	0.0	0.0	0.0	0.0
Pancreas, neuroendocrine carcinoma (NEC)	14	10	0.0	0.0	0.0	9.1	0.0	0.0	10.0	0.0	0.0
**Tumors of hematopoietic and lymphoid tissues**											
Hodgkin Lymphoma	103	92	0.0	0.0	0.0	0.0	0.0	0.0	0.0	0.0	0.0
Non-Hodgkin Lymphoma	62	58	0.0	0.0	0.0	0.0	0.0	0.0	0.0	0.0	0.0
Small lymphocytic lymphoma, B-cell type (B-SLL/B-CLL)	50	47	0.0	0.0	0.0	0.0	0.0	0.0	0.0	0.0	0.0
Diffuse large B-cell lymphoma (DLBCL)	114	109	0.0	0.0	0.0	0.0	0.0	0.0	0.0	0.0	0.0
Follicular lymphoma	88	86	0.0	0.0	0.0	0.0	0.0	0.0	0.0	0.0	0.0
T-cell non-Hodgkin lymphoma	24	22	0.0	0.0	0.0	0.0	0.0	0.0	0.0	0.0	0.0
Mantle cell lymphoma	18	18	0.0	0.0	0.0	0.0	0.0	0.0	0.0	0.0	0.0
Marginal zone lymphoma	16	14	0.0	0.0	0.0	0.0	0.0	0.0	0.0	0.0	0.0
Diffuse large B-cell lymphoma (DLBCL) in the testis	16	16	0.0	0.0	0.0	0.0	0.0	0.0	0.0	0.0	0.0
Burkitt lymphoma	5	3	0.0	0.0	0.0	0.0	0.0	0.0	0.0	0.0	0.0
**Tumors of soft tissue and bone**											
Tenosynovial giant cell tumor	45	37	0.0	0.0	0.0	0.0	0.0	0.0	0.0	0.0	0.0
Granular cell tumor	53	36	0.0	0.0	0.0	0.0	0.0	0.0	0.0	0.0	0.0
Leiomyoma	50	45	0.0	0.0	0.0	0.0	0.0	0.0	0.0	0.0	0.0
Leiomyosarcoma	87	80	1.3	0.0	0.0	0.0	0.0	0.0	0.0	1.3	0.0
Liposarcoma	132	99	0.0	0.0	0.0	0.0	0.0	0.0	0.0	0.0	0.0
Malignant peripheral nerve sheath tumor (MPNST)	13	12	8.3	0.0	0.0	0.0	0.0	0.0	0.0	8.3	0.0
Myofibrosarcoma	26	23	0.0	0.0	0.0	0.0	0.0	0.0	0.0	0.0	0.0
Angiosarcoma	73	49	0.0	0.0	0.0	1.9	0.0	0.0	0.0	0.0	0.0
Angiomyolipoma	91	68	0.0	0.0	0.0	0.0	0.0	0.0	0.0	0.0	0.0
Dermatofibrosarcoma protuberans	21	13	0.0	0.0	0.0	0.0	0.0	0.0	0.0	0.0	0.0
Ganglioneuroma	14	12	0.0	0.0	0.0	0.0	0.0	0.0	0.0	0.0	0.0
Kaposi sarcoma	8	3	0.0	0.0	0.0	0.0	0.0	0.0	0.0	0.0	0.0
Neurofibroma	117	107	0.0	0.0	0.0	0.0	0.0	0.0	0.0	0.0	0.0
Sarcoma, not otherwise specified (NOS)	75	63	1.5	0.0	0.0	0.0	0.0	0.0	0.0	1.6	0.0
Paraganglioma	41	40	0.0	0.0	0.0	0.0	0.0	0.0	0.0	0.0	0.0
Ewing sarcoma	23	9	0.0	0.0	0.0	0.0	0.0	0.0	0.0	0.0	0.0
Rhabdomyosarcoma	7	5	0.0	0.0	0.0	0.0	0.0	0.0	0.0	0.0	0.0
Schwannoma	121	118	0.0	0.0	0.0	0.0	0.0	0.0	0.0	0.0	0.0
Synovial sarcoma	12	8	0.0	0.0	0.0	0.0	0.0	0.0	0.0	0.0	0.0
Osteosarcoma	43	27	0.0	0.0	0.0	0.0	0.0	0.0	0.0	0.0	0.0
Chondrosarcoma	38	9	0.0	0.0	0.0	0.0	0.0	0.0	0.0	0.0	0.0

A comparison with histopathological features of cancer aggressiveness and/or clinical follow-up data in 120 different tumor entities revealed only few statistical associations (Table 2). Both CK5 and CK6 expressions were linked to high grade, estrogen and progesterone receptor negativity, and “triple negativity” in breast cancer (*p* < 0.0001 each), grade/stage progression in urothelial cancer (*p* < 0.0001), and RAS mutations in colorectal cancer (*p* < 0.01). CK5 expression was also associated with nodal metastasis in urothelial cancer (*p* = 0.0007) while CK6 expression was linked to nodal metastasis in gastric and ovarian cancer (*p* < 0.05). The CK5 and CK6 expression status was unrelated to overall patient survival in breast cancer (Supplementary Fig. 2a–c) and recurrence-free survival in patients treated by cystectomy for their urothelial carcinomas (Supplementary Fig. 2e–g).

**Table 2:** CK5 and CK6 immunostaining and tumor phenotype

				**CK5**							**CK6**							
			*n*	Neg. (%)	Weak (%)	Mod. (%)	Strong (%)	*p*	*n*		Neg. (%)		Weak (%)		Mod. (%)		Strong (%)	*p*
**Breast cancer of no special type**	Tumor stage	pT1	454	92.3	3.1	1.3	3.3	0.2082	479		84.1		7.3		5.8		2.7	0.5290
		pT2	351	90.0	2.8	1.4	5.7		370		81.6		8.1		6.2		4.1	
		pT3-4	77	85.7	9.1	1.3	3.9		82		89.0		3.7		6.1		1.2	
	Grade	G1	141	98.6	1.4	0.0	0.0	< 0.0001	151		91.4		5.3		3.3		0.0	< 0.0001
		G2	442	95.7	1.6	1.4	1.4		467		85.7		6.0		6.4		1.9	
		G3	325	80.0	7.1	2.5	10.5		344		75.9		10.8		6.7		6.7	
	Nodal stage	pN0	364	90.7	2.2	1.9	5.2	0.5639	379		83.6		8.2		4.5		3.7	0.9611
		pN1	185	89.7	4.3	1.1	4.9		199		82.4		9.0		5.0		3.5	
		pN2	57	94.7	1.8	1.8	1.8		64		84.4		6.3		7.8		1.6	
		pN3	45	86.7	6.7	0.0	6.7		46		82.6		10.9		4.3		2.2	
	HER2 status	Negative	736	90.1	3.7	1.8	4.5	0.5951	779		82.4		6.9		6.9		3.7	0.1808
		Positive	101	94.1	2.0	1.0	3.0		110		88.2		7.3		2.7		1.8	
	ER status	Negative	167	54.5	16.8	7.8	21.0	< 0.0001	185		54.1		18.4		11.9		15.7	< 0.0001
		Positive	633	99.7	0.3	0.0	0.0		662		90.2		4.2		5.1		0.5	
	PR status	Negative	327	76.8	8.3	4.0	11.0	< 0.0001	349		72.8		11.2		7.7		8.3	< 0.0001
		Positive	503	99.0	0.6	0.2	0.2		528		89.2		4.7		5.5		0.6	
	Triple negative	No	664	98.6	0.6	0.2	0.6	< 0.0001	699		89.0		5.0		5.3		0.7	< 0.0001
		Yes	115	41.7	21.7	10.4	26.1		126		44.4		20.6		14.3		20.6	
**Urinary bladder cancer**	Tumor stage	pTa G2 low	122	12.3	31.1	23.8	32.8	< 0.0001	134		86.6		6.7		4.5		2.2	< 0.0001
		pTa G2 high	90	27.8	27.8	25.6	18.9		104		75.0		14.4		7.7		2.9	
		pTa G3	92	51.1	17.4	17.4	14.1		102		79.4		14.7		3.9		2.0	
		pT2	125	40.8	7.2	7.2	44.8	0.2338	138		51.4		15.2		5.8		27.5	0.6770
		pT3	218	37.2	8.3	4.6	50.0		239		47.7		15.5		5.4		31.4	
		pT4	104	47.1	9.6	8.7	34.6		112		57.1		16.1		3.6		23.2	
	Nodal stage	pN0	268	32.5	9.3	7.1	51.1	0.0007	295		48.5		15.9		5.4		30.2	0.5191
		pN +	170	51.8	6.6	7.2	34.3		170		54.2		16.2		5.6		24.0	
**Endometroid endometrial carcinoma**	Tumor stage	pT1	93	59.1	28.0	9.7	3.2	0.2312	114		67.5		14.9		14.9		2.6	0.4008
		pT2	20	50.0	15.0	30.0	5.0		24		50.0		20.8		29.2		0.0	
		pT3-4	28	67.9	17.9	7.1	7.1		35		62.9		20.0		17.1		0.0	
	Nodal stage	pN0	46	60.9	21.7	15.2	2.2	0.9774	50		66.0		12.0		22.0		0.0	0.6323
		pN +	20	65.0	25.0	0.0	10.0		30		60.0		20.0		20.0		0.0	
				**CK5**						**CK6**								
			*n*	Neg. (%)	Weak (%)	Mod. (%)	Strong (%)	*p*	*n*	Neg. (%)		Weak (%)		Mod. (%)		Strong (%)		*p*
**Endometrioid ovarian cancer**	Tumor stage	pT1	21	52.4	28.6	14.3	4.8	0.4956	22	63.6		22.7		13.6		0.0		0.2097
		pT2	6	66.7	16.7	16.7	0.0		5	60.0		0.0		20.0		20.0		
		pT3	3	66.7	0.0	0.0	33.3		4	50.0		25.0		0.0		25.0		
	Nodal stage	pN0	21	57.1	28.6	9.5	4.8	0.7389	21	57.1		28.6		14.3		0.0		0.0281
		pN1	6	50.0	16.7	16.7	16.7		6	50.0		0.0		16.7		33.3		
**Serous ovarian cancer**	Tumor stage	pT1	25	56.0	20.0	12.0	12.0	0.6695	28	60.7		32.1		7.1		0.0		0.4721
		pT2	30	66.7	26.7	3.3	3.3		40	47.5		50.0		2.5		0.0		
		pT3	193	63.2	23.3	4.7	8.8		223	61.4		33.2		4.5		0.9		
	Nodal stage	pN0	61	72.1	19.7	1.6	6.6	0.2458	73	61.6		38.4		0.0		0.0		0.0244
		pN1	125	59.2	24.0	5.6	11.2		143	58.0		34.3		6.3		1.4		
**Stomach cancer**	Tumor stage	pT1-2	49	100.0	0.0	0.0	0.0	0.0663	48	85.4		12.5		0.0		2.1		0.2234
		pT3	90	87.8	5.6	4.4	2.2		89	77.5		11.2		5.6		5.6		
		pT4	87	92.0	3.4	1.1	3.4		87	80.5		6.9		6.9		5.7		
	Nodal stage	pN0	58	94.8	3.4	1.7	0.0	0.3701	54	88.9		9.3		1.9		0.0		0.0380
		pN +	167	91.0	3.6	2.4	3.0		168	78.0		9.5		6.0		6.5		
**Pancreatic adenocarcinomas**	Tumor stage	pT1	15	66.7	20	0	13.3	0.075	13	46.2		30.8		23.1		0		0.5751
		pT2	62	59.7	11.3	16.1	12.9		67	37.3		29.9		13.4		19.4		
		pT3	340	67.4	13.5	3.2	15.9		369	44.2		25.7		13.8		16.3		
		pT4	25	60	16	8	16		28	42.9		21.4		21.4		14.3		
	Grade	1	15	86.7	6.7	0	6.7	0.4984	16	68.8		18.8		6.3		6.3		0.2057
		2	313	65.2	14.4	5.1	15.3		334	44		26		13.5		16.5		
		3	95	61.1	13.7	6.3	18.9		106	34.9		27.4		18.9		18.9		
	Nodal stage	pN0	99	65.7	13.1	5.1	16.2	0.9962	102	41.2		30.4		12.7		15.7		0.7194
		pN +	342	66.1	13.5	5.3	15.2		374	43.9		24.9		15		16.3		
**Colon adenocarcinoma**	Tumor stage	pT1	56	100.0	0.0	0.0	0.0	0.3002	58	87.9		8.6		1.7		1.7		0.2232
		pT2	304	97.0	1.6	1.0	0.3		313	78.9		17.6		2.6		1.0		
		pT3	833	95.2	3.5	1.0	0.4		881	81.6		12.5		4.1		1.8		
		pT4	303	95.0	2.3	2.0	0.7		314	82.8		11.1		3.5		2.5		
	Nodal stage	pN0	786	95.4	3.1	1.1	0.4	0.9083	817	84.0		11.4		3.1		1.6		0.0675
		pN +	698	96.0	2.4	1.1	0.4		737	78.7		15.1		4.2		2.0		
	Vessel invasion	V0	1083	95.3	3.2	1.1	0.4	0.3112	1145	81.3		13.1		3.8		1.8		0.9808
		V +	390	96.9	1.5	1.0	0.5		400	82.3		12.5		3.5		1.8		
	Lymph invasion	L0	579	95.7	2.9	0.5	0.9	0.0559	596	81.9		12.8		3.4		2.0		0.8876
		L1	877	95.8	2.7	1.4	0.1		930	81.4		13.1		3.9		1.6		
	Tumor localization	Left colon	1110	96.4	2.0	1.2	0.5	0.0646	1159	83.2		11.7		3.5		1.6		0.0670
		Right colon	389	94.1	4.6	1.0	0.3		413	77.2		16.5		4.1		2.2		
	MMR status	Defective	83	100.0	0.0	0.0	0.0	0.0449	80	86.3		12.5		1.3		0.0		0.1937
		Proficient	1057	95.1	3.2	1.3	0.4		1106	80.7		13.9		3.7		1.7		
	RAS mutation status	Mutated	329	93.9	3.6	1.5	0.9	0.0054	343	75.2		17.2		4.7		2.9		0.0042
		Wildtype	434	98.2	0.9	0.9	0.0		446	85.2		9.9		3.6		1.3		

## Discussion

The successful analysis of a broad range of normal tissues and of more than 10,000 cancers for CK5 and CK6 by immunohistochemistry demonstrates important differences in the expression patterns of these cytokeratins. The data collected in this study suggest a superior diagnostic utility of monospecific CK5 or CK6 antibodies for immunohistochemical analysis as compared to bispecific CK5/6 antibodies. Additionally, literature data from various tumor types on CK5 expression (Supplementary Fig. 3) or CK6 expression (Supplementary Fig. 4) clearly demonstrate that such information cannot be easily obtained from the literature due to highly discrepant data across many studies.

That cytokeratins 5 and 6 have very different expression patterns is particularly demonstrated by the results of our extensive normal tissue analysis. Although both proteins were found in the majority of squamous epithelia, their staining patterns differed considerably with CK5 preferentially staining basal cells and CK6 preferably occurring in suprabasal layers. Given the complementary staining patterns of CK5 (basal) and CK6 (suprabasal), one might expect that the combined use of both antibodies could improve the positivity rate in squamous cell carcinomas of different sites of origin. That the combined analysis of CK5 and CK6 only increased the fraction of positive squamous cell carcinomas by 0–1% (average 0.1%) if compared to CK5 analysis alone and by 0–2% (average 0.5%) if compared to CK6 analysis alone does not provide strong evidence for superiority of using a combined CK5/6 antibody for the identification of squamous cell carcinomas, however.

The analysis of 109 non-squamous cell cancer tumor entities also did not suggest a particular rationale for combining anti-CK5 and anti-CK6 antibodies. The separate analysis of these antibodies showed for the vast majority of analyzed cancers that their CK5/6 positivity rate was largely driven by either CK5 or CK6 and that the addition of the other cytokeratin only minimally increased the positivity rate. Moreover, the positivity rate of both CK5 and CK6 was generally so low that the combination of both cytokeratins did still not result in a diagnostically useful information. Whether a tumor entity such as endometrioid carcinoma of the ovary is CK5 positive in 43% or CK5/6 positive in 49% does not impact the diagnostic information obtained by analyzing CK5 and/or CK6.

Especially in the case of thoracic tumors, the isolated CK5 analysis appears to be advantageous as compared to CK5/6 immunohistochemistry. For example, CK5 alone was positive in 100% of epithelioid mesotheliomas but in only 12.8% of lung adenocarcinoma and can thus be used in panels designed to distinguish these entities. Adding CK6 increases the positivity rate in lung adenocarcinoma to 23.7% and thus reduces the diagnostic potential for distinguishing mesothelioma from adenocarcinomas. CK6-positive adenocarcinomas also limit the utility of CK5/6 in the distinction of squamous cell carcinoma vs. adenocarcinoma of the lung which may be difficult and often requires the use of IHC panels. In another major application of CK5/6 IHC—the detection of basal cells in the prostate—CK5 is solely responsible for the beneficial effects, while CK6 is not staining any basal cells.

The availability of clinical follow-up data or histopathological data related to cancer aggressiveness enabled us to investigate the potential role of aberrant CK5 and CK6 expressions in 8 different cancer entities. The findings in bladder cancer further challenged the utility of combined CK5/6 analysis. In normal urothelium, CK5 is expressed in the basal cell layers. Because this basal layer staining is retained in the vast majority of non-invasive urothelial carcinomas, especially if they are of low grade, most pTa grade 1/2 tumors were scored as “weak” or “moderately” CK5 positive in our scoring system. In pTa grade 3 and especially in muscle-invasive urothelial carcinomas, the tumors often either completely lose CK5 expression or show CK5 expression in all cells. As a result, the fraction of CK5 negative and of strongly positive cases increased markedly with bladder cancer grade and stage. A link between CK5 expression and bladder cancer progression was earlier reported by several authors [38–41]. Most of these studies have employed antibodies against CK5/6 and it has been assumed that CK5/6 positivity reflects a “basal-type” molecular subgroup of urothelial carcinoma [42, 43]. Our separate analysis of CK6 revealed, however, that CK6 is unrelated to urothelial basal cells. CK6 upregulation has been considered a feature of squamous cell differentiation in urothelial cancer by others [44, 45].

That both CK5 and CK6 expressions were statistically linked to high grade in breast cancer is consistent with data from various earlier studies [46–48]. Accordingly, CK5/6 expression is an established feature of basal-type breast cancer which is well known for its poor clinical outcome [49]. These findings may reflect a general phenomenon. Multiple studies have described a tendency towards a poor prognosis and/or unfavorable tumor phenotype in cancers that show de novo expression of a cytokeratin which is not expressed in its normal cell type of origin [50–55]. An altered expression pattern of intermediate filaments appears to represent a common feature of cancer cell dedifferentiation that occurs during cancer progression and will thus be linked to unfavorable tumor features [56]. These significant associations with relevant histological tumor aspects were not found in endometrial, ovarian, stomach, pancreatic, and colon cancers, and argue against a major role of CK5 and CK6 expressions for tumor progression. The relationship between CK5 and CK6 expression and KRAS remains unclear as direct or indirect interactions between these proteins are not known and also not expected based on the individual functions of these two proteins.

In summary, our data show that important properties which are commonly attributed to CK5/6 antibodies such as basal cell staining in the prostate, distinction of mesothelioma and squamous cell carcinoma from adenocarcinoma of the lung, and identification of basal-type features in urothelial cancer are solely driven by CK5. At least for the purpose of distinguishing thoracic tumors, monospecific CK5 antibodies may be better suited than bispecific CK5/6 antibodies in diagnostic immunohistochemistry.

## Supplementary Information

Below is the link to the electronic supplementary material.

Supplementary file1 Relative importance of CK5 and CK6 in different tumor types. a) Tumor types with a predominant role of CK6. b) Tumor types with a predominant role of CK5. Squamous cell carcinomas are shown in both diagrams. (PDF 28 KB)

Supplementary file2 Prognostic relevance of CK5, CK6 or the joint analysis of CK5 and CK6 in a-c) breast cancers of no special type and d-f) muscle invasive urinary bladder cancers. (PDF 38 KB)

Supplementary file3 Graphical representation of CK5 data from this study (marked by an “x”) in comparison with the previous literature. Red dots are used for studies involving 2–10 cases, orange dots are used for studies involving 11–25 cases, and green dots are used for studies involving >25 cases. (PDF 28 KB)

Supplementary file4 Graphical representation of CK6 data from this study (marked by an “x”) in comparison with the previous literature. Red dots are used for studies involving 5 cases, green dots are used for studies involving >25 cases. Literature data [30] do not disclose the histological subtype of gastric cancers. (PDF 23 KB)

## Data Availability

Raw data are available upon reasonable request. All data relevant to the study are included in the article.

## Declarations

The usage of archived diagnostic left-over tissues for manufacturing of TMAs and their analysis for research purposes as well as patient data analysis has been approved by local laws (HmbKHG, §12,1) and by the local ethics committee (Ethics Commission Hamburg, WF-049/09). All work has been carried out in compliance with the Helsinki declaration.
