# Supplementary figures and images for: Cytokeratin 5 and cytokeratin 6 expressions are unconnected in normal and cancerous tissues and have separate diagnostic implications
Source: Virchows Arch. 2021 Sep 24;480(2):433–47. doi: 10.1007/s00428-021-03204-4 (PMC8986736; doi:10.1007/s00428-021-03204-4)

a)

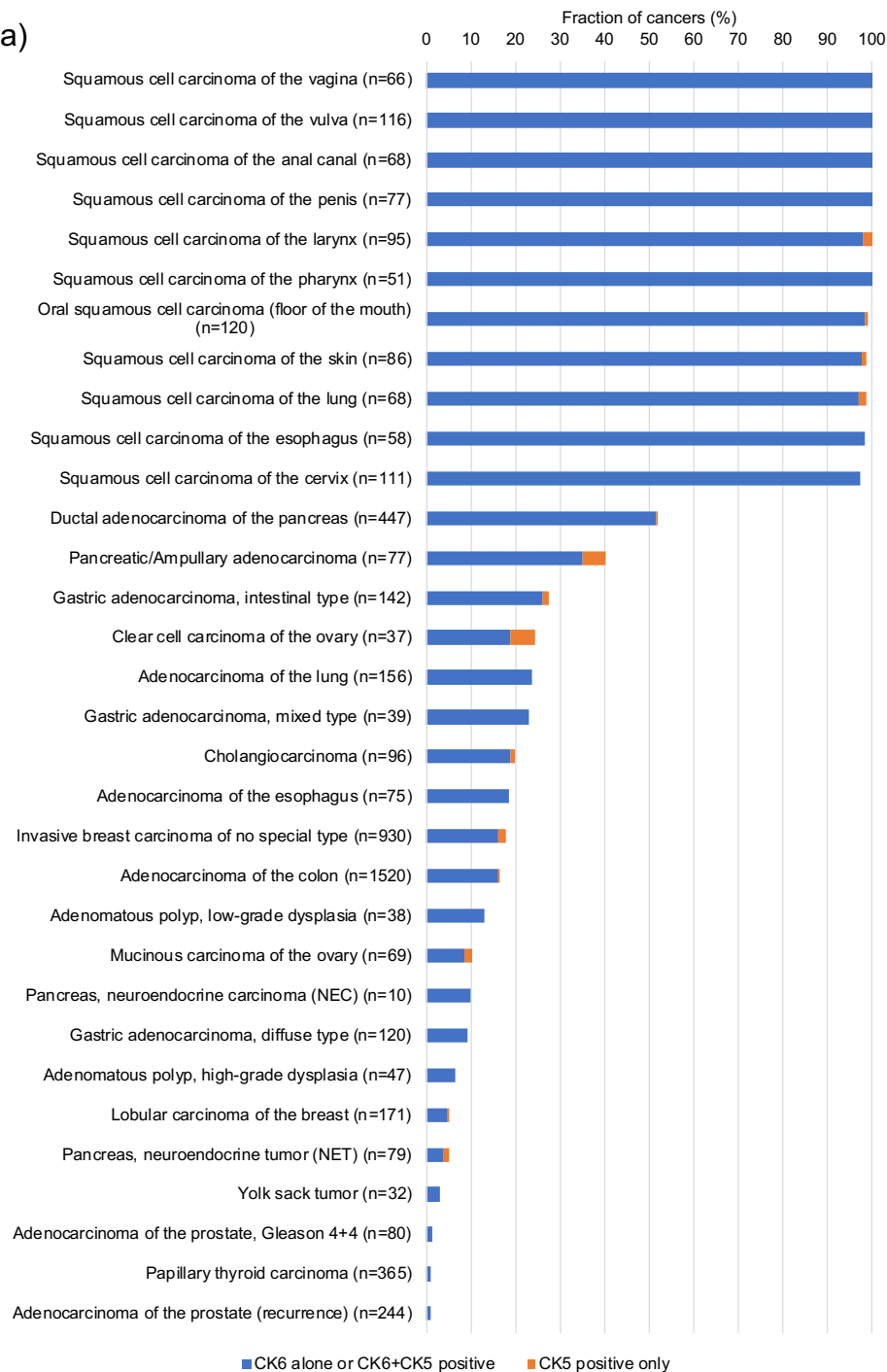

b)

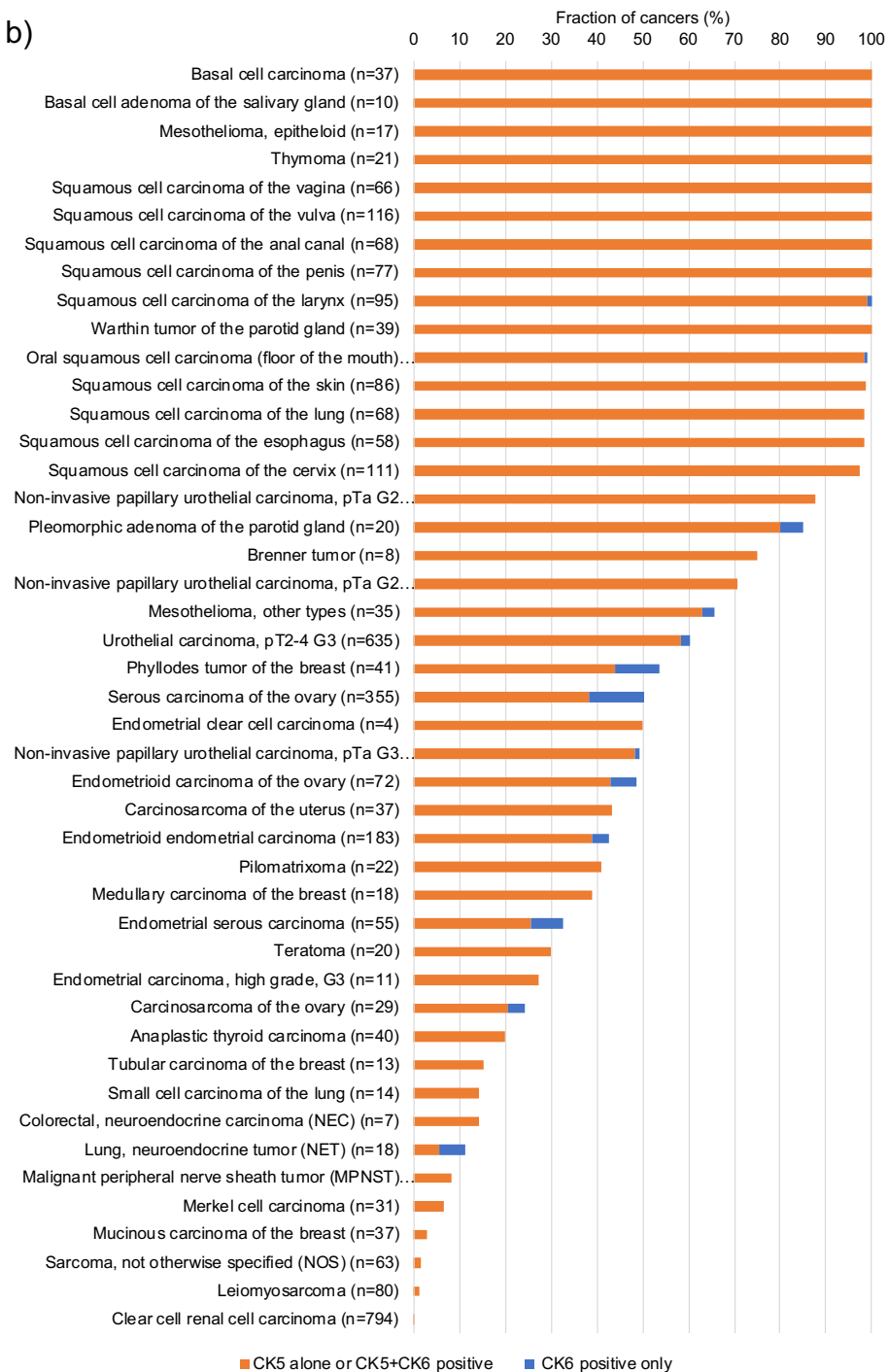

Supplement: Supplementary file 1 — Supplementary file1 Relative importance of CK5 and CK6 in different tumor types. a) Tumor types with a predominant role of CK6. b) Tumor types with a predominant role of CK5. Squamous cell carcinomas are shown in both diagrams. (PDF 28 KB) [file 428_2021_3204_MOESM1_ESM.pdf]

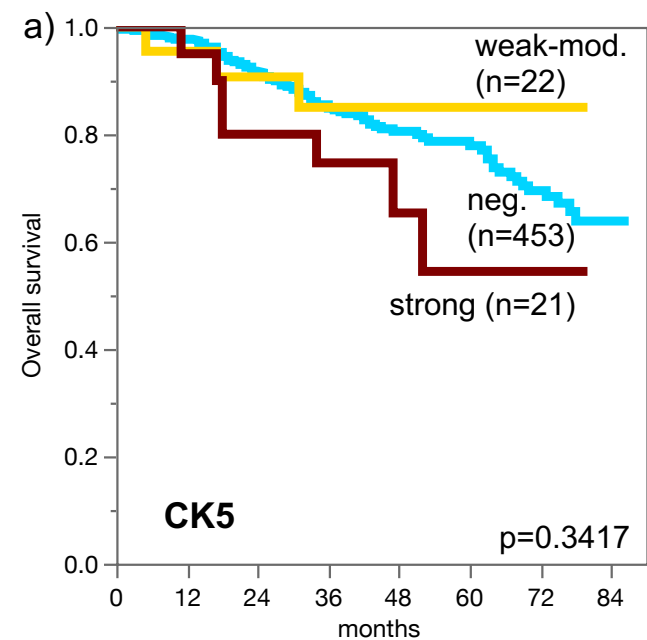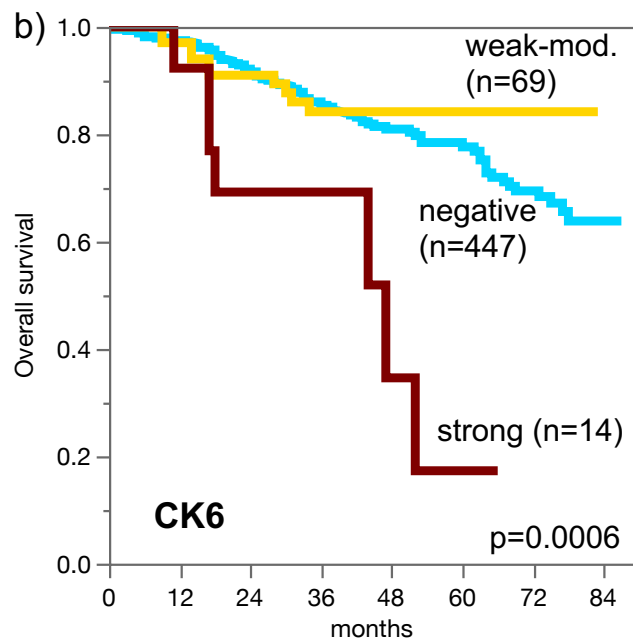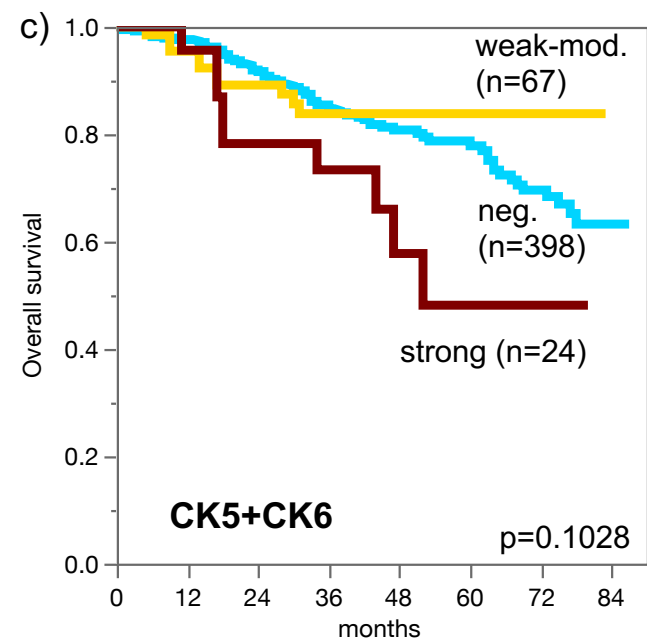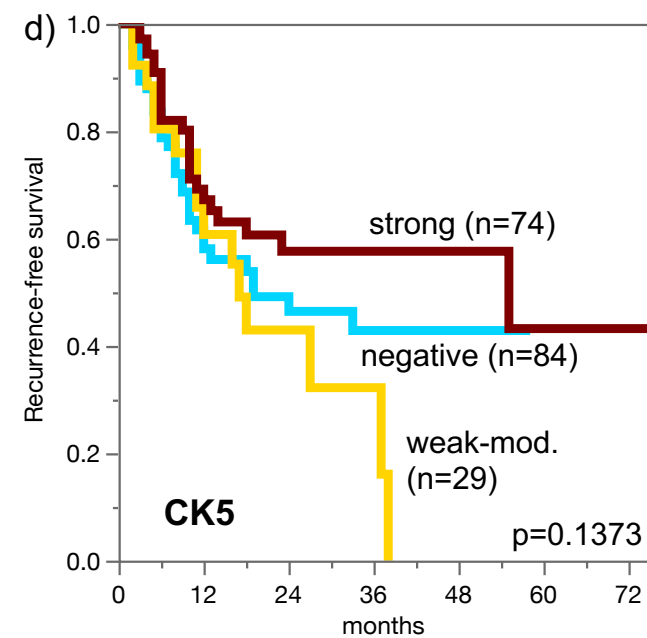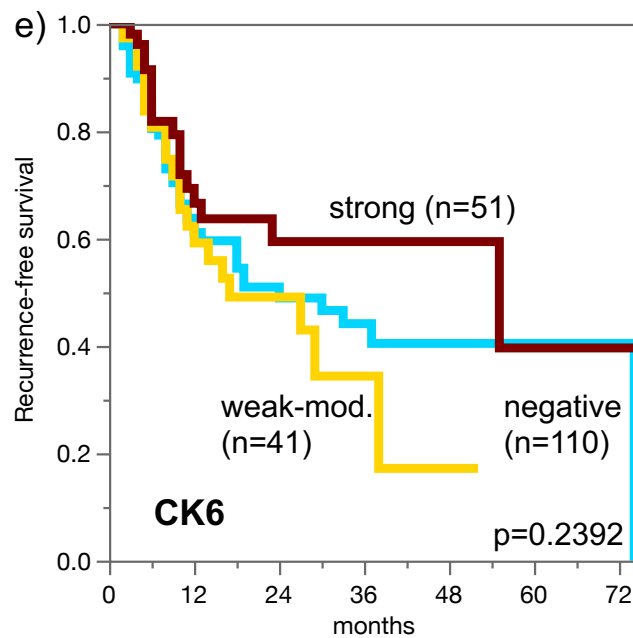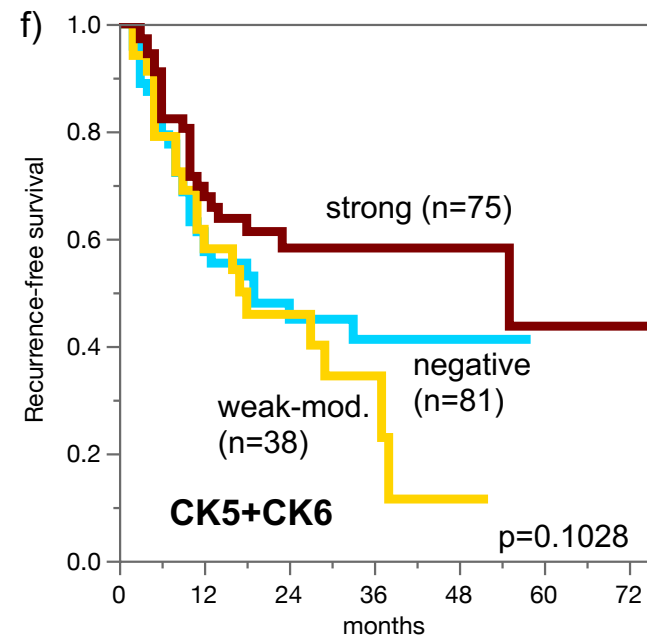

Supplement: Supplementary file 2 — Supplementary file2 Prognostic relevance of CK5, CK6 or the joint analysis of CK5 and CK6 in a-c) breast cancers of no special type and d-f) muscle invasive urinary bladder cancers. (PDF 38 KB) [file 428_2021_3204_MOESM2_ESM.pdf]

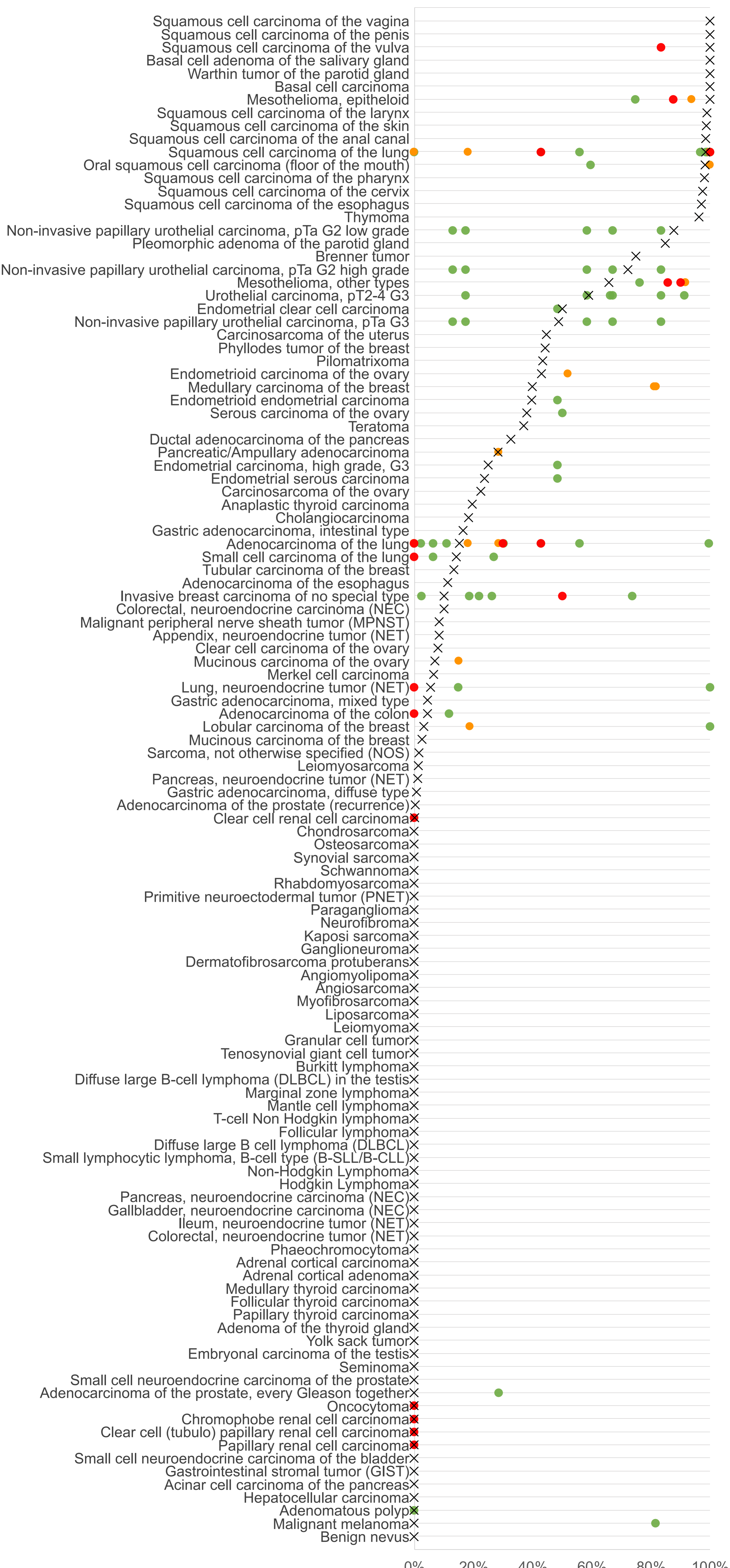

Supplement: Supplementary file 3 — Supplementary file3 Graphical representation of CK5 data from this study (marked by an “x”) in comparison with the previous literature. Red dots are used for studies involving 2–10 cases, orange dots are used for studies involving 11–25 cases, and green dots are used for studies involving >25 cases. (PDF 28 KB) [file 428_2021_3204_MOESM3_ESM.pdf]

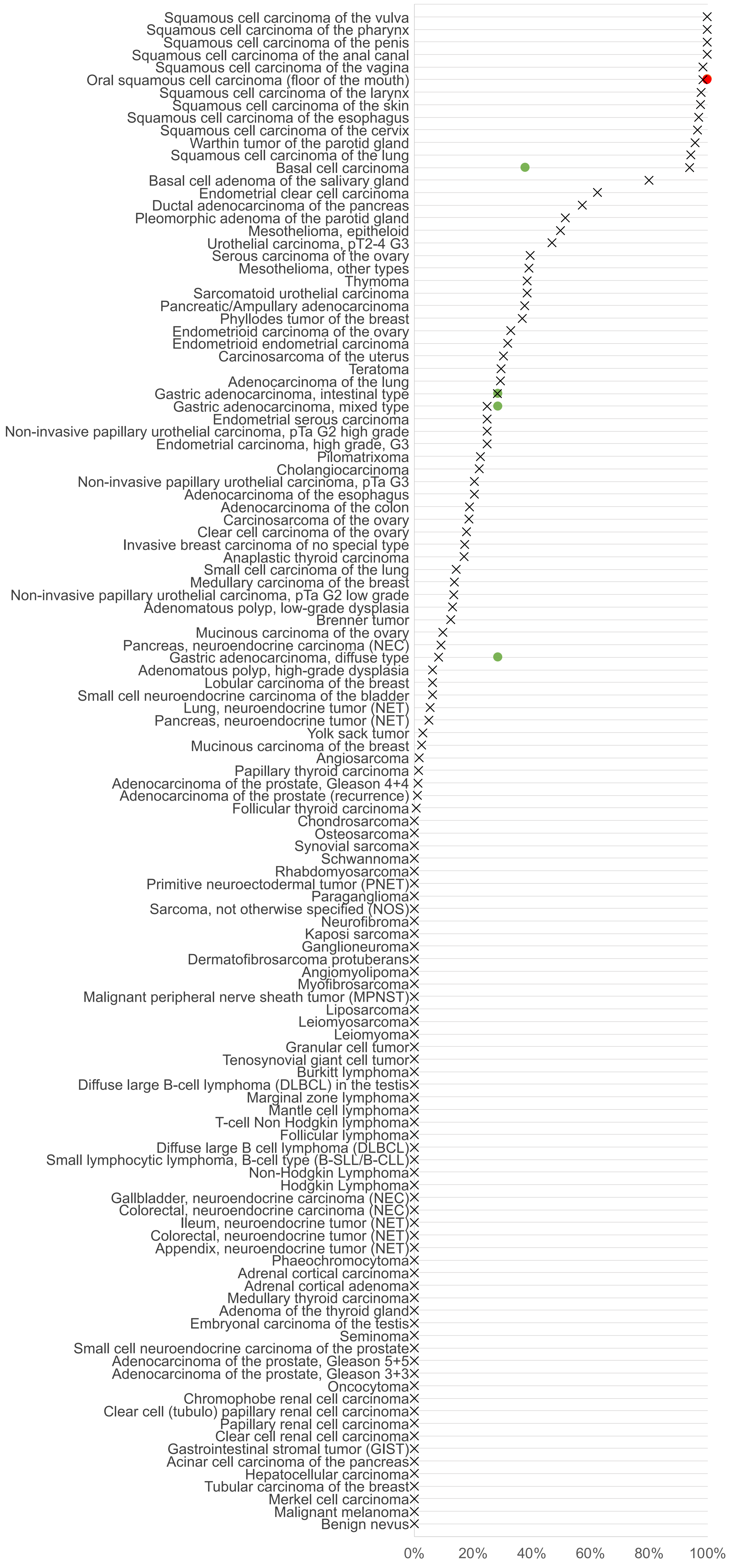

Supplement: Supplementary file 4 — Supplementary file4 Graphical representation of CK6 data from this study (marked by an “x”) in comparison with the previous literature. Red dots are used for studies involving 5 cases, green dots are used for studies involving >25 cases. Literature data [30] do not disclose the histological subtype of gastric cancers. (PDF 23 KB) [file 428_2021_3204_MOESM4_ESM.pdf]
